# Supplementary material for: GWAS for Early-Establishment QTLs and Their Linkage to Major Phenology-Affecting Genes (Vrn, Ppd, and Eps) in Bread Wheat
Source: Genes (Basel). 2023 Jul 24;14(7):1507. doi: 10.3390/genes14071507 (PMC10378780; doi:10.3390/genes14071507)

Supplementary Table 1: List of *QTLs* controlling multiple traits in different planting times

QTLs	**Chr**	**Total *QTL* Interval**	**Traits**	**Most Significant Marker(s)**
QMpt.bisa.1B.3	1B	0.52	TGW, PG_BTH	1B_572013120, 1B_572077620
QMpt.bisa.1D.2	1D	0.11	PG_GFD, FLGLFW	1D_30541993, 1D_30542016
QMpt.bisa.1D.3	1D	0.59	PG_GFD, PG_BTH	1D_31127895
QMpt.bisa.2A.1	2A	1.27	SR, FLGLFL	2A_31950515, 2A_32942025, 2A_33177689
QMpt.bisa.2A.3	2A	315.42	PG_GFD, FLGLFW, SR, TGW, PG_DTB, HUS	2A_154952478, 2A_186427483, 2A_188487886, 2A_202884383, 2A_218333140, 2A_220203137, 2A_223189397, 2A_232886081, 2A_233356485, 2A_234393958, 2A_235357606, 2A_236151345, 2A_237924432, 2A_265043237, 2A_269930695, 2A_274288787, 2A_276745579, 2A_285841892, 2A_308438332, 2A_310110750, 2A_310348491, 2A_312397332, 2A_315761862, 2A_318828556, 2A_319326184, 2A_320703902, 2A_330767318, 2A_364159450, 2A_370794256, 2A_381365384, 2A_413685276, 2A_415317868, 2A_416350269,
QMpt.bisa.2A.6	2A	0.82	PG_GFD, FLGLFL	2A_754163560, 2A_754601516
QMpt.bisa.2B.2	2B	0.49	SR, PG_DTB, PG_BTH	2B_59220162, 2B_59220243, 2B_59584538
QMpt.bisa.2D.3	2D	94.10	EGC, PG_GFD, PG_BTH, FLGLFW	2D_141998244, 2D_224333851, 2D_226305009, 2D_259407805, 2D_325652495, 2D_336112223, 2D_372937863
QMpt.bisa.3A.4	3A	39.54	EGC, GRYLD	3A_525879700, 3A_533589765, 3A_557212913, 3A_560829823, 3A_561026957, 3A_561851147
QMpt.bisa.3A.5	3A	4.74	PG_BTH, PG_GFD	3A_653959431, 3A_654785037
QMpt.bisa.3B.2	3B	34.99	PG_DTB, CTIR	3B_420519725, 3B_453434148
QMpt.bisa.4B.2	4B	7.78	PG_DTB, TGW	4B_496823471, 4B_498390062, 4B_504307823
QMpt.bisa.4B.6	4B	0.28	PG_DTB, SR	4B_656452095
QMpt.bisa.5A.1	5A	16.83	PG_DTB, PG_GFD	5A_67475215, 5A_70136376
QMpt.bisa.5A.3	5A	3.62	EGC, LFGLFL, HUS, PG_BTH, PG_GFD, TGW	5A_579216178, 5A_579315291, 5A_579849967, 5A_580467995, 5A_581738768, 5A_581738776
QMpt.bisa.5B.1	5B	29.85	PG_BTH, FLGLFL, SR	5B_396108086, 5B_397181195, 5B_397682314, 5B_400100474, 5B_400454479, 5B_410694082, 5B_415524761, 5B_421473320, 5B_421719134

QTLs	Chr	Total *QTL* Interval	Traits	Most Significant Marker(s)
QMpt.bisa.5B.2	5B	20.30	PG_DTB, PG_BTH, PG_GFD, FLGLFL	5B_552860736, 5B_554810601, 5B_557927509, 5B_561282306, 5B_562371212, 5B_565985738, 5B_566674836, 5B_567647671, 5B_568515593, 5B_568678349, 5B_569083742, 5B_569889755, 5B_570824048, 5B_571199142, 5B_571213548
QMpt.bisa.5B.3	5B	0.10	PG_DTB, SR	5B_576445730, 5B_576445786
QMpt.bisa.5B.4	5B	0.436909	PG_DTB, SR, GRYLD	5B_582834253, 5B_582974222, 5B_589459465, 5B_589896374
QMpt.bisa.5B.5	5B	6.19	PG_DTB, SR	5B_591184260, 5B_591830635, 5B_592792409, 5B_593016847, 5B_593137406, 5B_594155067, 5B_594614262, 5B_594913947, 5B_595649946, 5B_596648484
QMpt.bisa.5D.1	5D	0.97	PG_DTB, FLGLFL, SR	5D_544153906, 5D_544217347, 5D_544799149
QMpt.bisa.6A.1	6A	4.77	PG_GFD, HUS	6A_61080142, 6A_61212031, 6A_61215999, 6A_61400594, 6A_61422653, 6A_61817777, 6A_62571452, 6A_63109075, 6A_63193128, 6A_63205035, 6A_65844428
QMpt.bisa.6A.3	6A	387.25	PG_DTB, PG_GFD, HUS, FLGLFL, FLGLFW, CTIR, SR	6A_107243402,6A_109489944,6A_110621637,6A_111503215,6A_112907140,6A_113174486,6A_114457003,6A_187733247,6A_208514838,6A_209963947,6A_215325817,6A_232059246,6A_232059265,6A_272380641,6A_312709201,6A_327439351,6A_333494073,6A_353882239,6A_368885626,6A_378567041,6A_395513261,6A_414252855,6A_420669060,6A_427422144,6A_430583146,6A_439553873,6A_451070801,6A_493731895
QMpt.bisa.6A.5	6A	0.000027	GRYLD, FLGLFL	6A_609057074
QMpt.bisa.6B.2	6B	489.57	PG_BTH, PG_GFD, TGW, FLGLFL, HUS, CTIR, GRYLD	6B_154004312, 6B_169795390, 6B_193075087, 6B_207880142, 6B_208115717, 6B_211211877, 6B_213003566, 6B_299348988, 6B_303643410, 6B_356925185, 6B_363538527, 6B_363772854, 6B_383909822, 6B_384505441, 6B_413515478, 6B_447927068, 6B_451403111, 6B_451403123, 6B_455175520, 6B_458132601, 6B_459114835, 6B_471674109, 6B_476732821, 6B_563977112, 6B_587174528, 6B_592698766, 6B_595952036, 6B_596935914, 6B_604804854, 6B_623993849, 6B_624022505, 6B_640984096

QTLs	Chr	Total *QTL* Interval	Traits	Most Significant Marker(s)
QMpt.bisa.6D.2	6D	6.93	EGC, FLGLFL	6D_5945276, 6D_6343707, 6D_7993836, 6D_12018637
QMpt.bisa.6D.3	6D	55.86	EGC, FLGLFL	6D_178681816, 6D_229305823
QMpt.bisa.7A.6	7A	225.55	PG-BTH, HUS, FLGFLF, CTIR, SR, TGW	7A_246722996, 7A_272835142, 7A_274007853, 7A_274990066, 7A_281995484, 7A_305699834, 7A_367700921, 7A_375024289, 7A_382005083, 7A_385044692, 7A_423788356, 7A_429976050, 7A_432535332, 7A_435236652, 7A_436695555, 7A_438167392, 7A_454649603, 7A_459209377, 7A_471725086
QMpt.bisa.7B.3	7B	235.86	PG_DTB, EGC, CTIR, SR	7B_113442605, 7B_117141590, 7B_177787916, 7B_337375747
QMpt.bisa.7B.5	7B	1.70	PG_GFD, HUS, EGC	7B_669973991, 7B_670542596, 7B_671427054
QMpt.bisa.7D.2	7D	3.19	PG_DTB, PG_GFD, FLGLFL, SR, TGW	7D_55486522, 7D_55529933, 7D_55920290, 7D_56296098, 7D_57246447, 7D_57775472, 7D_57903956, 7D_58046109, 7D_58206696, 7D_58486999, 7D_58493344, 7D_58500973, 7D_58633321
QMpt.bisa.7A.4	7A	6.23	GRYLD, PG_DTB	7A_68211963, 7A_68774152, 7A_69311409, 7A_69937982, 7A_70208197, 7A_70893713, 7A_71591808, 7A_69311409, 7A_70893713

Supplementary Table 2: The utilisation of a detailed designation or appellation results in a clear and unequivocal communication of the subject matter. Multiple codes are employed in the research to enhance readability for the audience. The codes utilised in the study have been expounded upon in detail in the subsequent table.

**Abbreviations**	**Full form**
↑	Positive Selection Gain
↓	Negetive Selection Gain
AD	Average Deviation
APSIM	The Agricultural Production Systems sIMulator
BISA	Borlaug Institute for South Asia
BLUP	Best Linear Unbiased Prediction
bp	Base pair
CENEB	Campo Experimental Norman E. Borlaug
CI	Confidnece Interval of mean
CIMMYT	International Maize and Wheat Improvement Centre
cM	Centimorgan
CTIR	Canopy Temperature Increasing Rate
DAYSMT	Days to Maturity
DNA	Deoxy ribonucleic acid
DTB	Days to Booting
DTHD	Days to Heading
EGC	Early Ground Cover
EP	Early Planting
Eps	earliness per se gene
FA	Factor analysis
fD	Photoperiod factor
FLGLFA	Flag Leaf Area
FLGLFL	Flag Leaf Length
g	Gram
GBS	Genotyping by Sequencing
GDD	Growing Degree Days
GFD	Grain Filling Duration
GRYLD	Grain Yield
GWAS	Genome Wide Association Studies
HUS	Height Upto Spike
IGP	Indo -Gangetic plain
IRT	Infrared Thermometer
IWGSC	International Wheat Genome Sequencing Consortium
LAI	Leaf Area Index
LD	Iinkage Diseqilibriam
LR	Leaf Rust
LRTg	Likelihood ratio for genotypes ()
LRTge	Likelihood ratio for genotype and planting time interaction ()
MAS	Marker Assisted Selection
Mbp	Mega base pair

Abbreviations	Full form
ME	Mega-environment
MET	Multi-environmental
mg	Milligram
MGIDI	Multi-trait Genotype-Idiotype Distance Index
ml	Milliliter
mm	Millimeter
MPE	Mean Performance and Stability
MR	Moderately Resistant
MS	Moderately Susceptible
NAM	Nested Connection Mapping
NARS	National Agricultural Research System
NDVI	Normalized Difference Vegetation Index
NGS	Next-generation Sequencing
NIC-GoI	National Informatics Centre-Government of India
nm	Nanometer
NPK	Nitrogen:Phosporus:Potassium
ºC	Degree Celsius
PG_BTH	Photo-Growing Degree Days for Height Upto Spike
PG_DAYSMT	Photo-Growing Degree for Days Days to Maturity
PG_DTB	Photo-Growing Degree Days for Days to Booting
PG_DTHD	Photo-Growing Degree Days for Days to Heading
PG_GFD	Photo-Growing Degree Days for Grain Filling Duration
PGDD	Photo-Growing Degree Days
PH	Plant Height
Ppd	Photoperiod sensetivity gene
QTL	Quantitative Trait Loci
R	Resistant
REML	Restricted Residual Maximum Likelihood
S	Susceptible
S1	Season 1
S2	Season 2
S3	Season 3
SAARC	South Asian Association for Regional Cooperation
SABWGPYT	South Asia Bread Wheat Genomic Prediction Yield Trial
SI	Selection Intensity
SNP	Single Nucleotide Polymorphism
SpkLng	Spike Length
SR	Stem Rust
SR	Senescence Rate
SSR	Simple Sequence Repeat
TGW	Thousand Grain Weight
Tmax	Maximum Temperature
Tmin	Minimum Temperature

**Abbreviations**	**Full form**
Topt	Optimum Temperature
TP	Timely Planting
Vrn	Vernalization gene
WEDD	Degree Days Estimated by Wang and Engel, 1998
YR	Yellow Rust

<u>Supplementary Figure 1</u>: Weather parameter for three seasons at BISA farm, Ludhiana

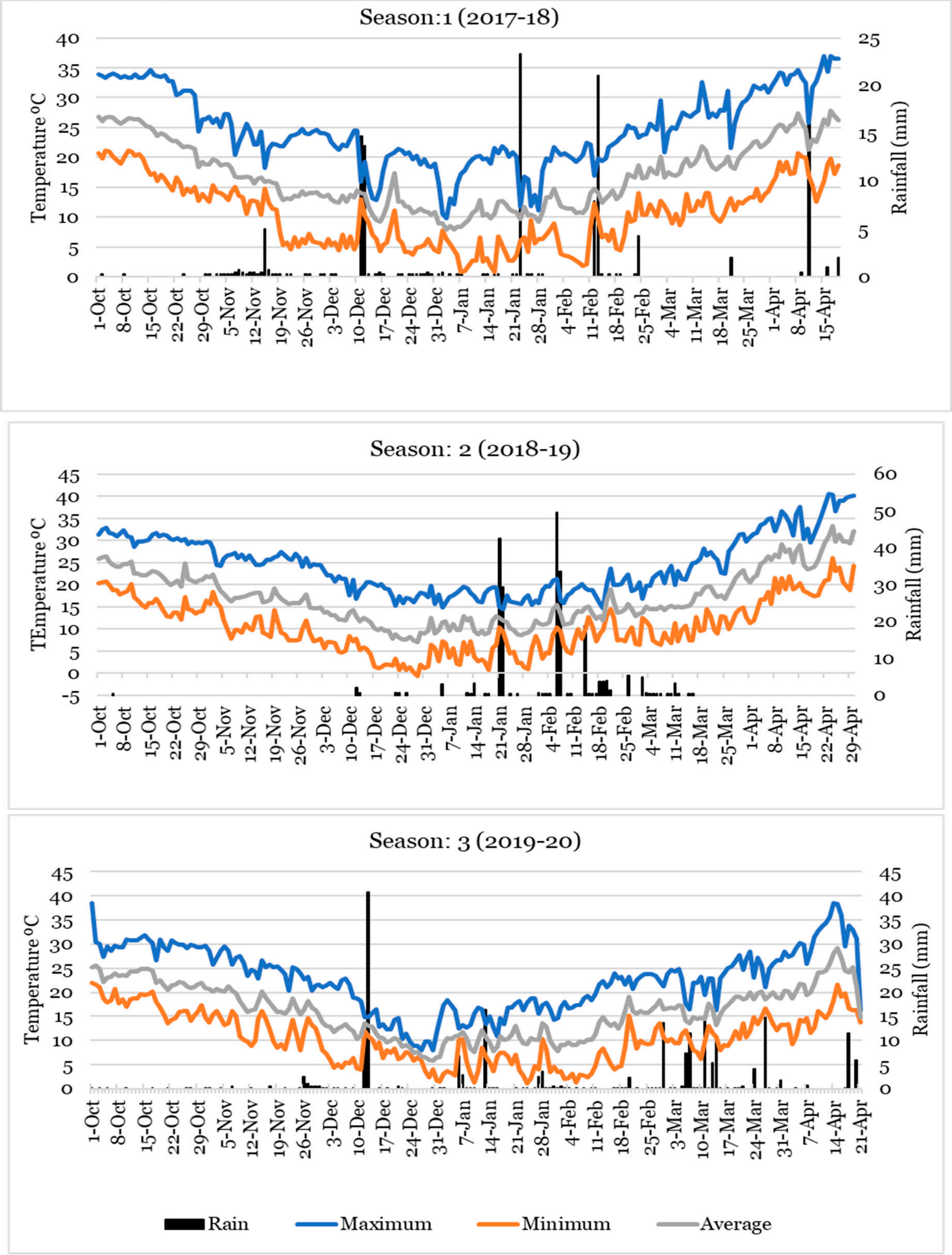

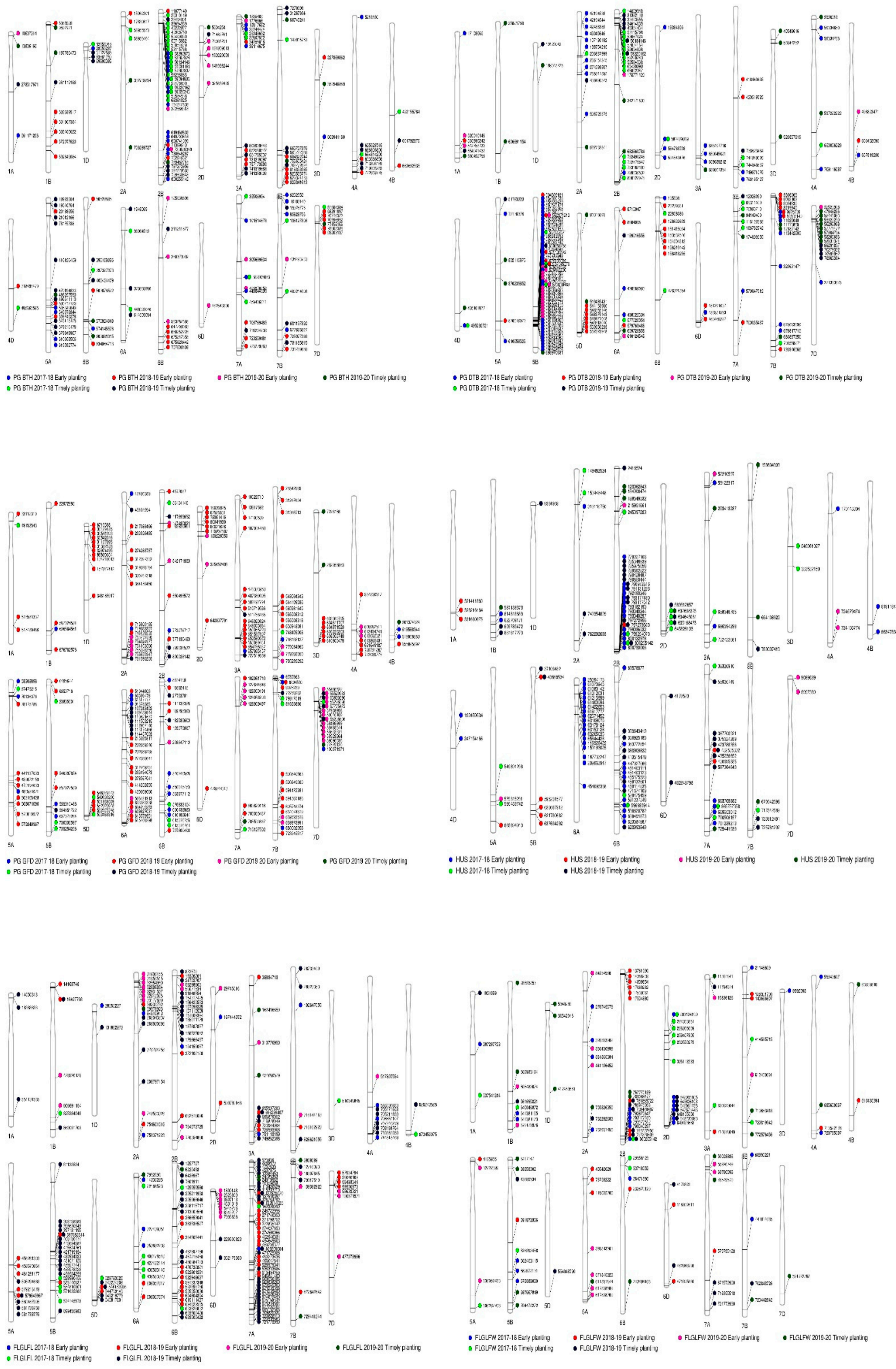
PG BTH 2017-18 Early planting
PG BTH 2018-19 Early planting
PG BTH 2019-20 Early planting
PG BTH 2019-20 Timely planting
PG BTH 2017-18 Timely planting
PG BTH 2018-19 Timely planting
PG DTB 2017-18 Early planting
PG DTB 2018-19 Early planting
PG DTB 2019-20 Early planting
PG DTB 2019-20 Timely planting
PG DTB 2017-18 Timely planting
PG DTB 2018-19 Timely planting
PG GFD 2017-18 Early planting
PG GFD 2018-19 Early planting
PG GFD 2019-20 Early planting
PG GFD 2019-20 Timely planting
PG GFD 2017-18 Timely planting
PG GFD 2018-19 Timely planting
HUS 2017-18 Early planting
HUS 2018-19 Early planting
HUS 2019-20 Early planting
HUS 2019-20 Timely planting
HUS 2017-18 Timely planting
HUS 2018-19 Timely planting
FLGLFL 2017-18 Early planting
FLGLFL 2018-19 Early planting
FLGLFL 2019-20 Early planting
FLGLFL 2019-20 Timely planting
FLGLFL 2017-18 Timely planting
FLGLFL 2018-19 Timely planting
FLGLFW 2017-18 Early planting
FLGLFW 2018-19 Early planting
FLGLFW 2019-20 Early planting
FLGLFW 2019-20 Timely planting
FLGLFW 2017-18 Timely planting
FLGLFW 2018-19 Timely planting

a

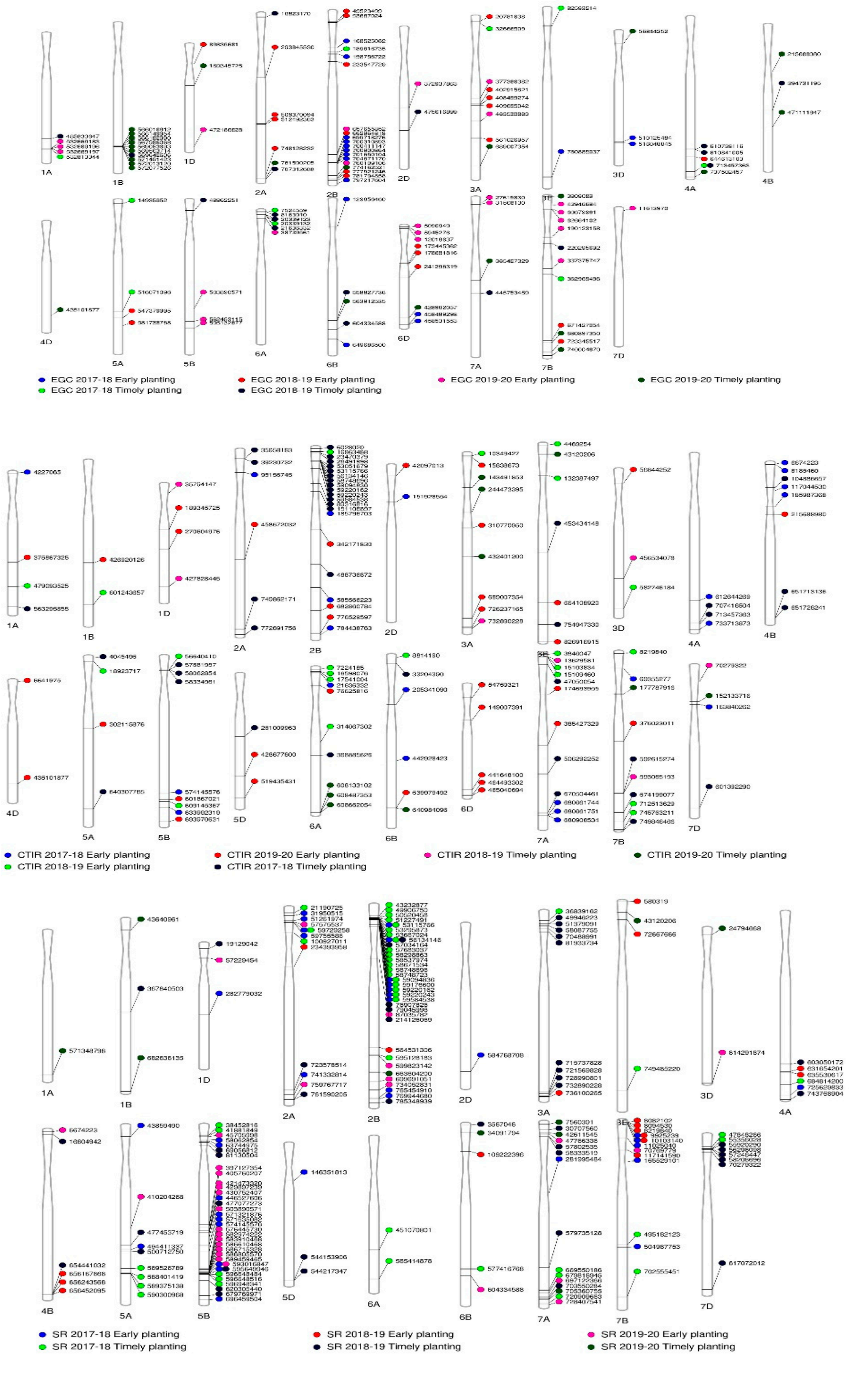
EGC 2017-18 Early planting
EGC 2017-18 Timely planting
EGC 2018-19 Early planting
EGC 2018-19 Timely planting
EGC 2019-20 Early planting
EGC 2019-20 Timely planting
CTIR 2017-18 Early planting
CTIR 2018-19 Early planting
CTIR 2019-20 Early planting
CTIR 2017-18 Timely planting
CTIR 2018-19 Timely planting
CTIR 2019-20 Timely planting
SR 2017-18 Early planting
SR 2017-18 Timely planting
SR 2018-19 Early planting
SR 2018-19 Timely planting
SR 2019-20 Early planting
SR 2019-20 Timely planting

b

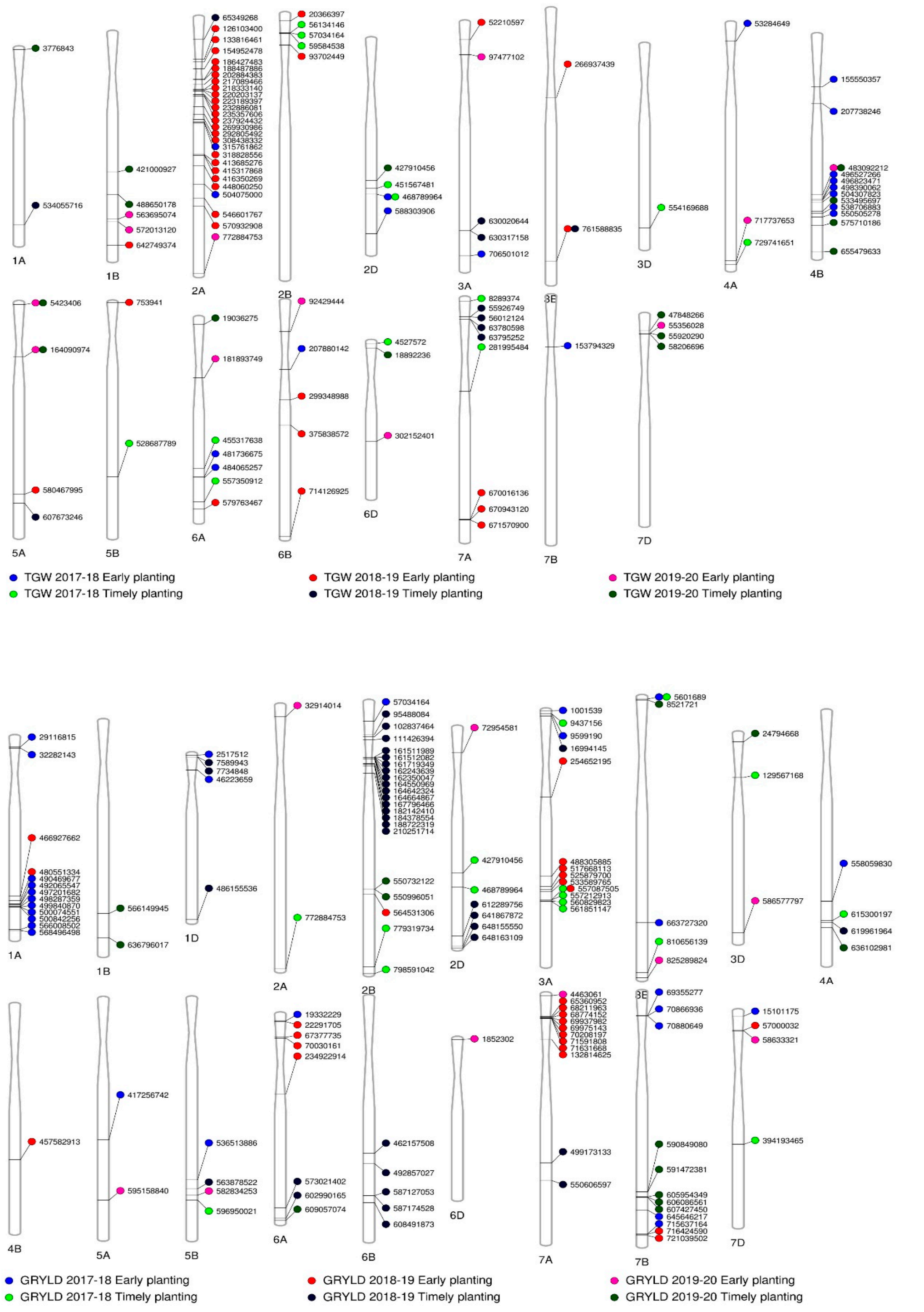

c

<u>Supplementary Figure 2</u>: Positions of the significant markers in the reference genome for; (a) phenological traits, (b) physiological traits, and (c) TGW and grain yield

Supplementary Figure 3a: Number of significant markers across the genome identified through GWAS

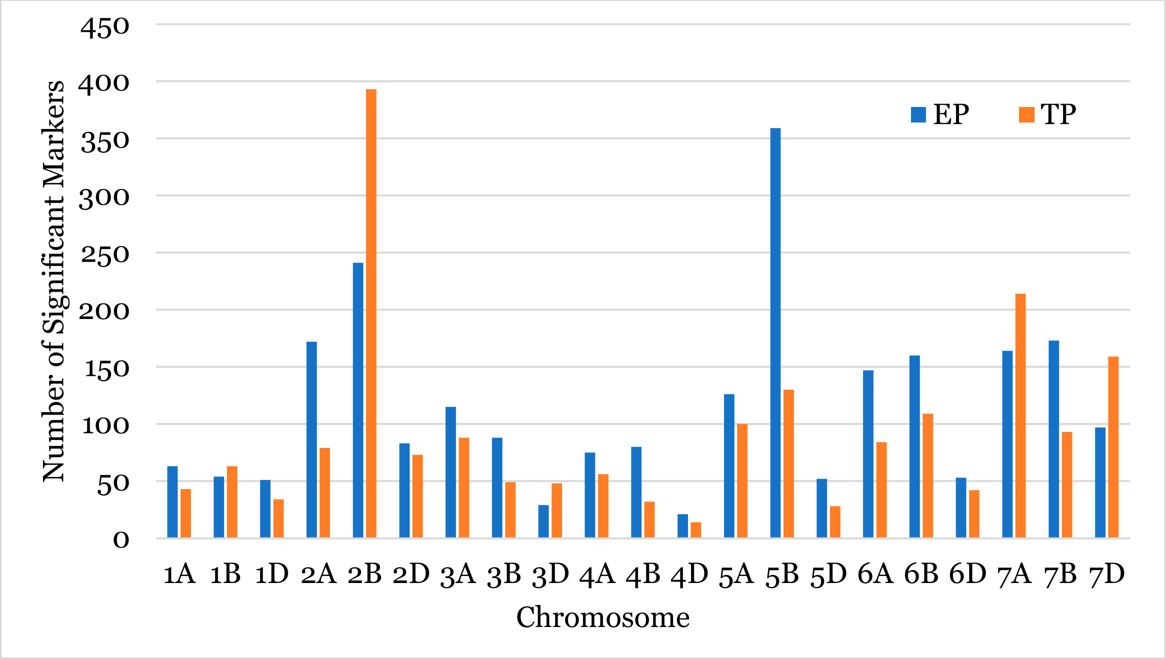

Supplementary Figure 3b: Genome wise QTL numbers showing their effect on planting times

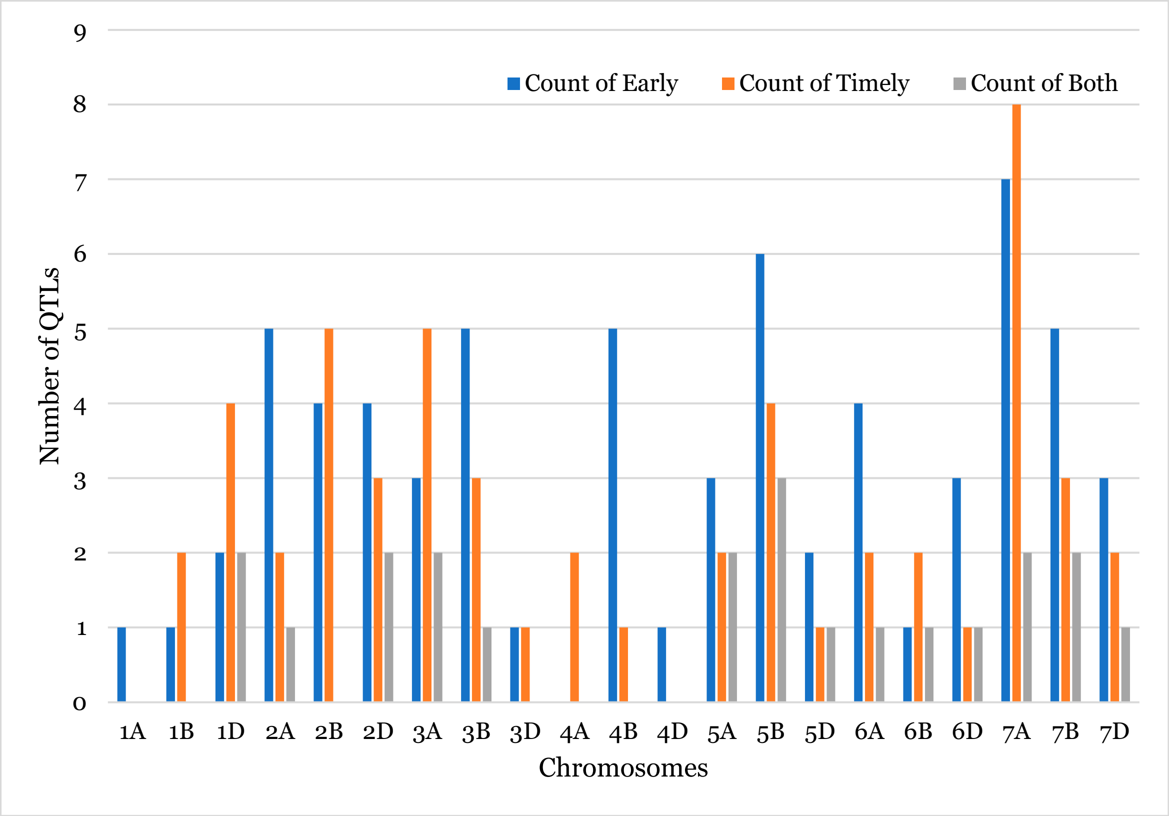

Supplementary Figure 4: Manhattan plots showing several markers are significantly associated with traits of interests studied in three consecutive seasons for early and timely planting

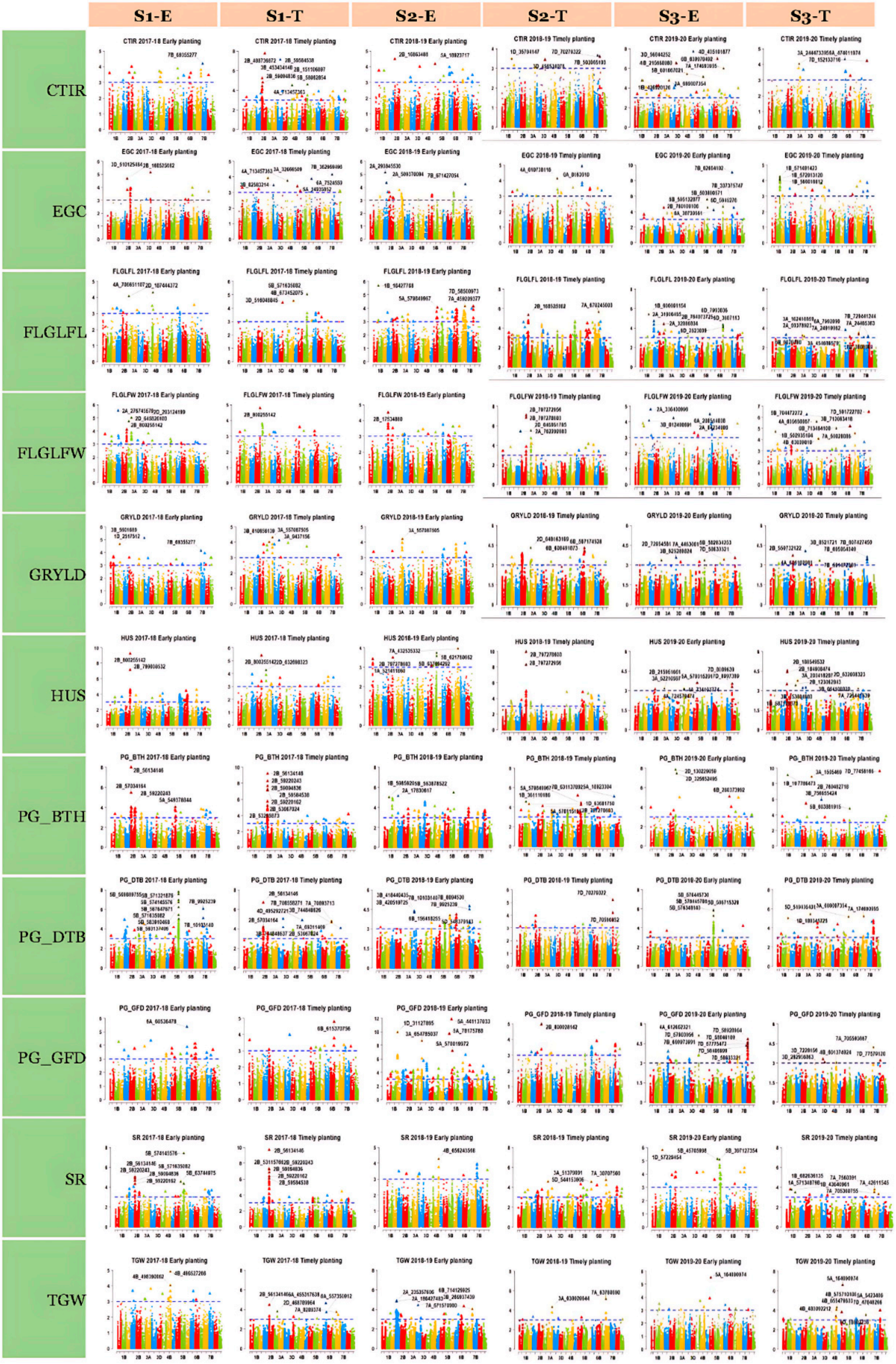

Supplementary Figure 5: Two decades (1998-2017) of historical temperature data revealed that there is minor high temperature in October which is the proposed early planting time for India

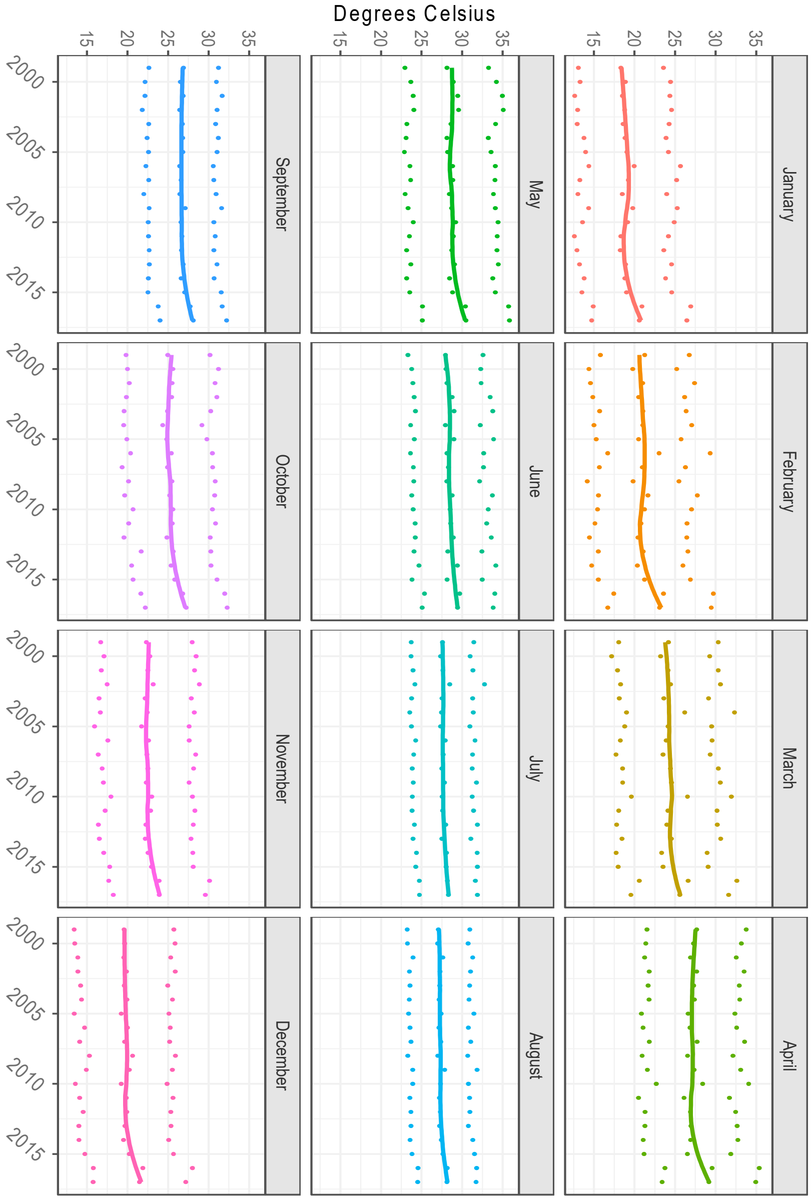

Supplementary Figure 6: Pearson correlation confidence Interval (Corr_CI) of traits in early and timely planting. The estimated range of correlation at a confidence level of 95% showed that the trait's correlation had varied significantly in shifting planting time.

a) Phenological traits with plant stature

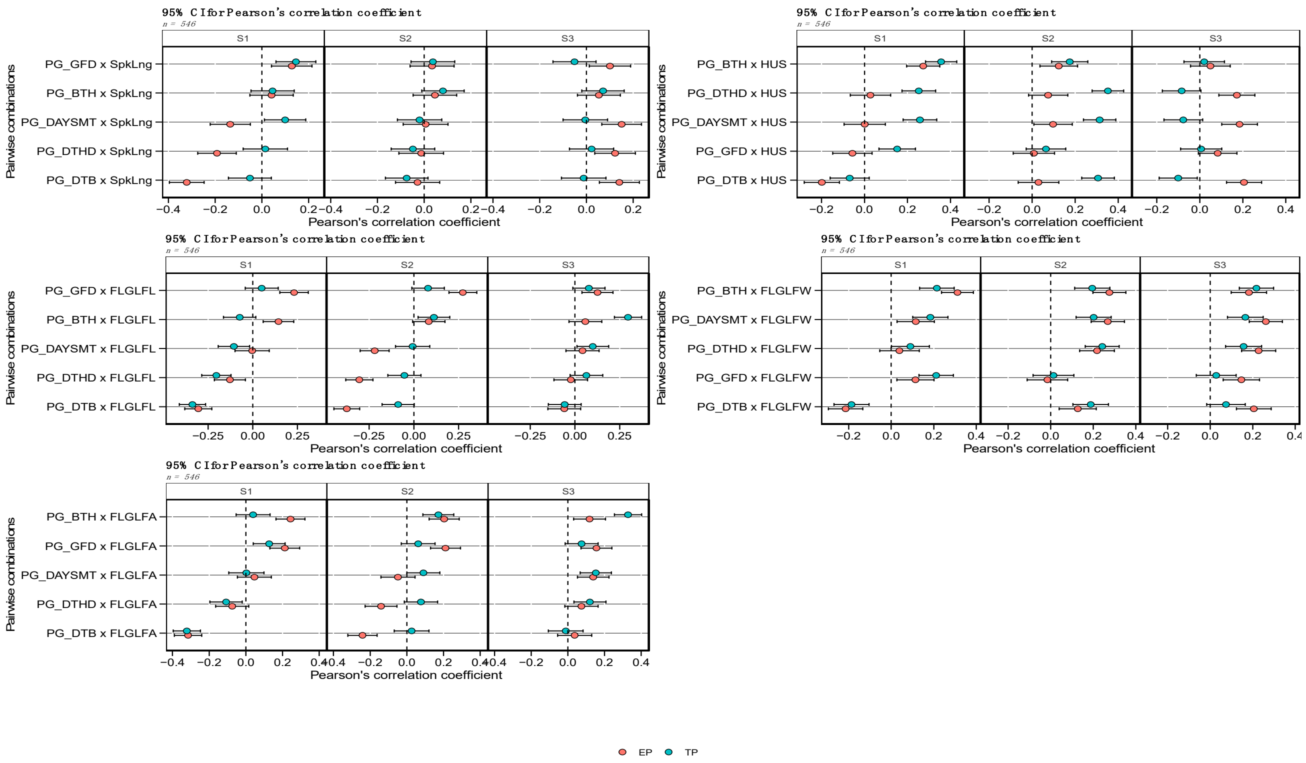

b) Phenological traits with physiological traits

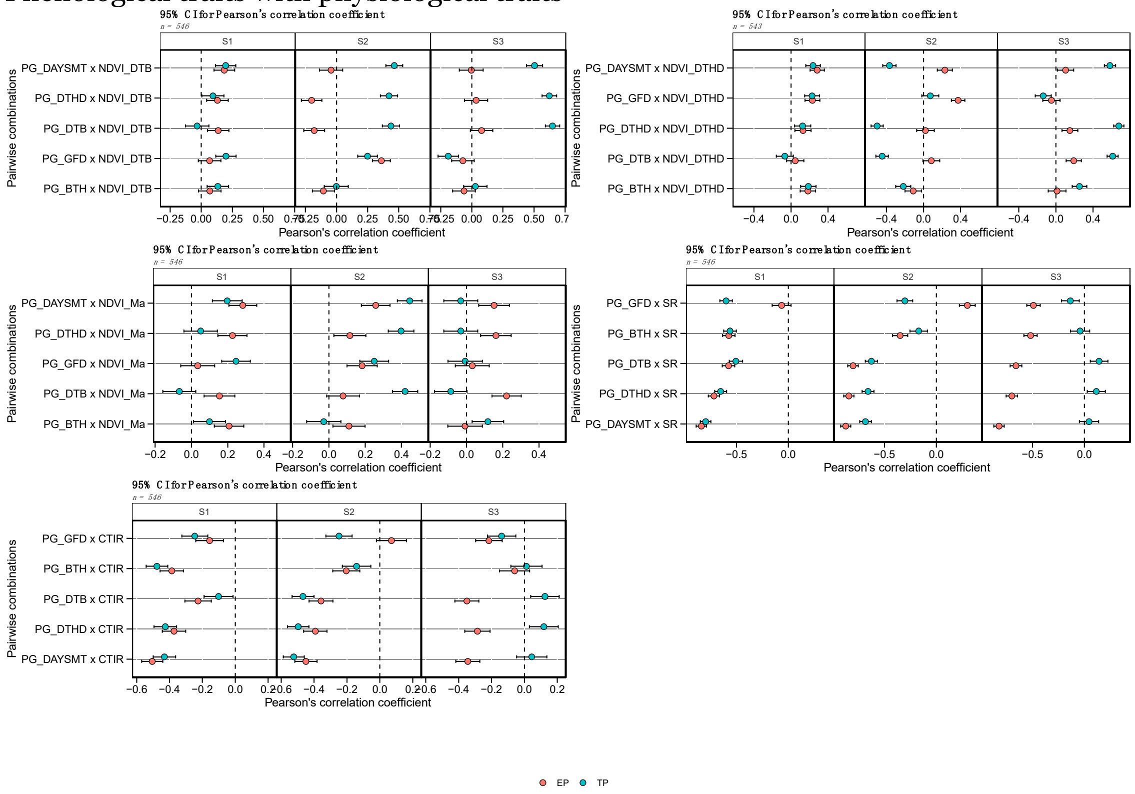

c) Physiological traits with plant stature

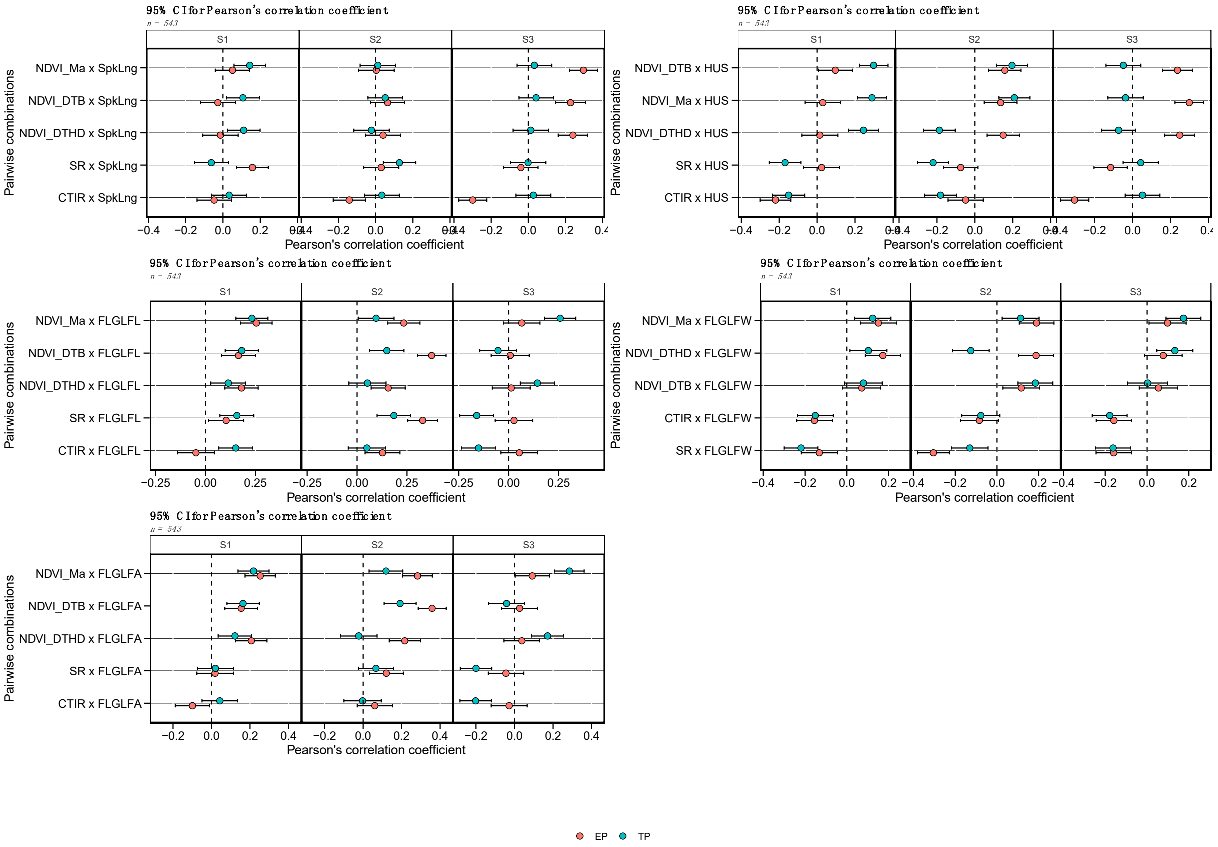

d) All the traits with TGW and grain yield

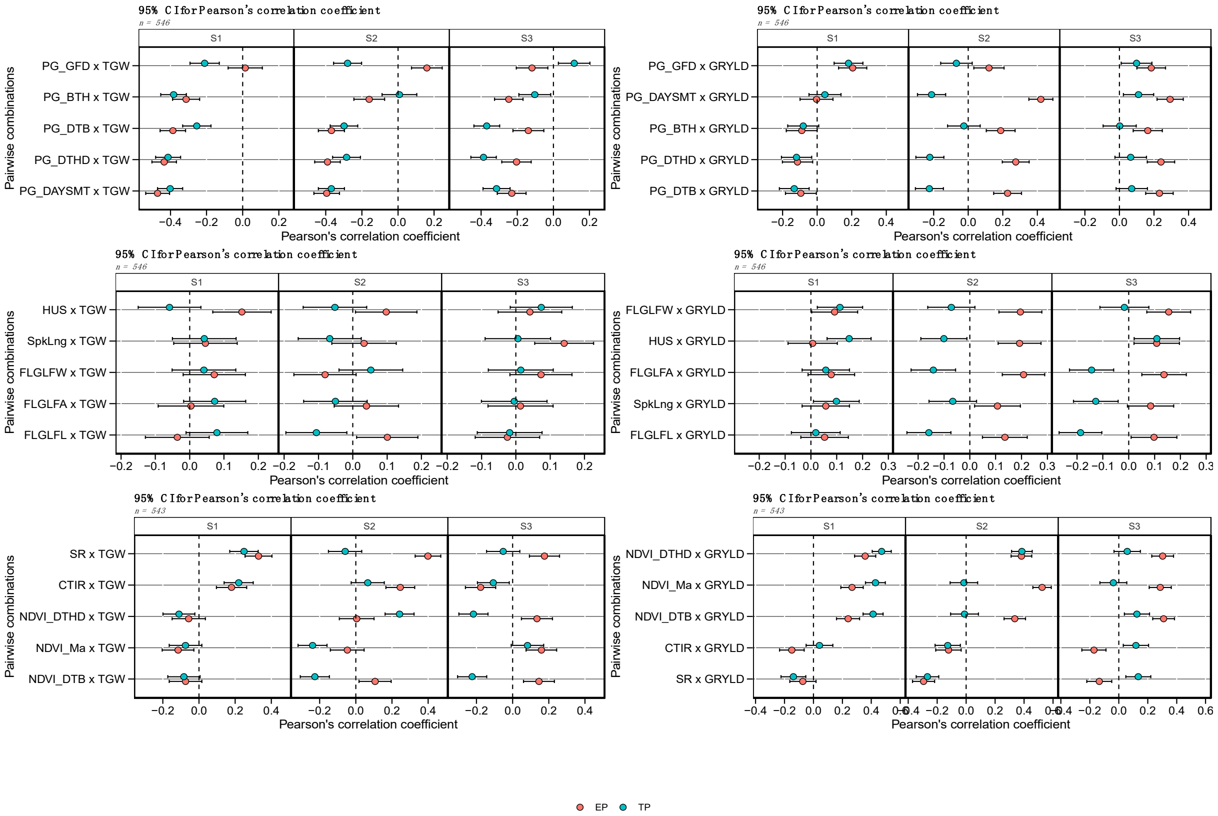

Supplement: Supplementary file 1 [file genes-14-01507-s001.zip › genes-2414093-supplementary.pdf]
